# Burden of Disease and Unmet Needs in the Diagnosis and Management of Atopic Dermatitis in the Arabic Population of the Middle East

**DOI:** 10.3390/jcm12144675

**Published:** 2023-07-14

**Authors:** Omar Mahmoud, Gil Yosipovitch, Enas Attia

**Affiliations:** 1 Dr. Phillip Frost Department of Dermatology and Cutaneous Surgery, Miami Itch Center, Miller School of Medicine, University of Miami, Miami, FL 33136, USA; oxm219@med.miami.edu; 2 Department of Dermatology, Venereology and Andrology, Ain Shams University Hospitals, Cairo 11566, Egypt; 3 Department of Dermatology, Ain Al Khaleej Hospital, Abu Dhabi 88206, United Arab Emirates

**Keywords:** atopic eczema, dermatitis, allergy, itch, skin disease, treatment, prevention, epidemiology, ethnic differences, cellular, molecular, immunological, physiological therapeutic, sociocultural mechanisms

## Abstract

Atopic dermatitis (AD) affects diverse ethnic groups with significant disparities in prevalence, disease progression, clinical outcomes, and access to care. There are limited data on AD in the Arabic population of the Middle East, yet there is a substantial economic and psychosocial burden of AD in this region with a large unmet need with regards to disease management that is critical to address. There is a trend of increasing prevalence of AD in the Arab Middle East; however, due to the large environmental, socioeconomic, and sociocultural heterogeneity of this region, prevalence varies greatly across and within countries. Similarly, clinical differences in disease presentations exist across the region, although data are limited. In this review, we will present clinical phenotypes of AD common in different regions of the Arab Middle East, and data on prevalence, genetic variations, and challenges of treatment. Further studies exploring molecular biomarkers, genetic polymorphisms, immune factors, and the microbiome of patients in the region will help to elucidate the mechanism behind ethnic differences in AD in this population as well as to understand susceptibilities and treatment response.

## 1. Introduction

Atopic dermatitis (AD) is a chronic inflammatory skin disease characterized by recurrent eczematous skin lesions and intense itching. Worldwide, it affects 10–30% of children and 2–10% of adults, with a two- to three-fold increase in prevalence observed over the last several decades [1].

The Arab Middle East is the geopolitical region commonly encompassing the Arabian Peninsula (Arabian Gulf region and Yemen), and the Arabic countries around the southern and eastern shores of the Mediterranean Sea (Syria, Lebanon, Palestine, Jordan, and Egypt). The Arabian Gulf region comprises the countries that extend along the western coastline on the Arabian (Persian) Gulf. These countries are Iraq, Kuwait, Qatar, Bahrain, Saudi Arabia, United Arab Emirates, and Oman. The Arab Middle East region has seen a growing burden of AD, but despite this, the region remains underrepresented in AD literature. Underdiagnosis and a lack of regionally appropriate diagnostic and research tools contribute to the scarcity of information. An investigation into the nature and underlying causes of this burden can help address challenges to medical care in this region and expand our understanding of AD. In this article, we aim to provide an overview of the epidemiology, potential mechanisms, distinctive clinical features, and burden of AD in the Arab Middle East region, with an emphasis on recommendations for improving its management in this part of the world.

## 2. Epidemiology

Over the last decade, the political and territorial fragmentation of many Arab Middle Eastern countries has led to migration of their nationals to other neighboring countries, particularly high-income countries in the Arabian Gulf region; the two most obvious cases are Syria and Yemen, as well as Libya and Iraq to a lesser extent. Thus, most of the countries in the Arabian Gulf region are experiencing demographic imbalances as a result, with expatriate labor constituting most of the population, ranging from 37% in Saudi Arabia to 89% in the UAE. In other words, the citizens themselves are becoming a minority across the Gulf region. Previous epidemiologic studies in this region have hardly specified whether the study population consists of citizens, expatriate residents, or a mixture of both.

Historically, the prevalence of AD throughout the Arab Middle East has been lower than North America and Europe; however, AD prevalence is increasing in the region due to many factors potentially including increasing urbanization and pollution [2]. Due to the large environmental, socioeconomic, and sociocultural heterogeneity of the Arab Middle East, prevalence varies greatly across and within countries.

Reliable diagnostic criteria for AD are essential. The Hanifin and Rajka diagnostic criteria, proposed over 30 years ago, summarized the four main characteristics when assessing a patient with suspected AD: pruritus, distribution and typical morphology, chronic or recurrent symptoms, and personal or family history of asthma, rhinitis, and/or dermatitis. International Study of Asthma and Allergies in Childhood (ISAAC) criteria evaluated the prevalence of asthma and other allergic conditions among children living in different regions of the world, using a core questionnaire, addressing the presence of itchy rash in a specific distribution with a relapsing nature and possible sleep disturbance. The questionnaire was translated from English into the languages spoken by the populations that were tested. A recent, multinational study by Silverberg et al. investigated the 12-month prevalence of diagnosed AD (meeting ISAAC criteria and having ever been told by a physician that they have eczema) in pediatric populations [3]. Among Middle Eastern countries included in the study, the overall prevalence of AD in the UAE was 16.7%, and in Saudi Arabia was 19.8% (Table 1). When only ISAAC criteria were considered, the overall prevalence of reported AD was higher than the diagnosed AD in the UAE (39.1%) and Saudi Arabia (37.1%). The prevalence of diagnosed AD was lower than AD prevalence based on ISAAC criteria alone, which the authors suggested may be due to misclassification of diseases that may have a similar presentation to AD [3]. Another recent large-scale epidemiologic AD study conducted by Maspero et al. reported the prevalence of AD in Egypt (3.6%), the UAE (11.6%), and Saudi Arabia (15.3%) [4].

Epidemiologic information from ISAAC phase 3 surveyed 1 million school children in the world between 1999 and 2004 and estimated the lifetime prevalence of AD in 6- and 7-year-old children in the Eastern Mediterranean region (including Egypt, Jordan, Lebanon, Libya, Palestine, and Syria) to be 7.2%. The global prevalence in this age range was 14.2%. Amongst 13- and 14-year-olds, the lifetime prevalence of AD was estimated to be 10.8% in the Eastern Mediterranean region, compared with a global prevalence of 12.8% for this age range. The ISAAC also reported the prevalence of AD in 13- to 14-year-old adolescents in Syria (3.9%), Kuwait (11.3%), and Oman (14.4%) [5,6]. In these large population studies, prevalence is reported to vary between and within countries in the Middle East [7].

Prevalence rates vary greatly within the same country depending on the study city and population. A 2008–2009 Saudi Arabian study conducted of 854 Taif citizens reported the prevalence of AD to be 45.4% [6]. In a recent study by Al-Frayh et al., the prevalence rates of AD in different Saudi Arabian cities, determined with questionnaire-based assessments, was found to be 43.5% in Hofuf, 32.6% in Riyadh, and 31.9% in Jeddah [8]. The overall prevalence of physician-diagnosed AD was found to be 12.5%. Nahhas et al. found that the prevalence of physician-diagnosed AD in Saudi Arabian children aged 6–12 years of age to be 14% [8]. 

Ibrahim et al. evaluated the prevalence of AD in 3436 school children in Dubai and the Northern emirates in the UAE and reported it to be 12.9% in children aged 6–7 years and 14.6% in children aged 13–14 years of age. These findings are comparable to previous studies by Al-Hammadi et al. and Behbehani et al. who reported a prevalence of diagnosed AD of 9% and 12%, respectively [9]. A UAE study by Al Hammadi et al. extracted data from the insurance e-claims data of the Dubai Real-World Database (DRWD) and found the prevalence of mild-to-moderate AD in this population to be 5.5%. Approximately 80% of the population of Dubai, a predominately expatriate community, is covered by private insurance, and thus, the DRWD captures nearly 100% of those who are covered under private insurance [10].

Ziayb et al. conducted a school-based cross-sectional study of adolescents 11–14 years of age across Kuwait and found the 12-month prevalence of patients reporting a history of ever being diagnosed by a doctor with eczema to be 19.5% (736 out of 3775 of participants) [11].

The ISAAC conducted a cross-sectional study of school children aged 11 to 14 years at Al Maadi and the Al Maasara region in Cairo, Egypt between February and April 2014, and of 308 students interviewed, the prevalence of AD was found to be 12.01% [12].

A Lebanese study of 5544 children and adolescents aged 5 to 14 years found the prevalence of AD to be 11.5% [13,14]. In a different study of 3909 children and adolescents aged 5 to 12 years old, the authors found the prevalence of AD to be 11.8% [15].

Regional and ethnic diversity may play a role in the variable prevalence rates seen in Middle Eastern countries [7]. However, the varying estimates of AD prevalence within and between countries make it difficult to extrapolate the effect of ethnicity on the risk of developing AD [16].

**Table 1:** Reported prevalence data of AD in the Middle East.

Country	Prevalence Range	Prevalence	Study	Study Information
United Arab Emirates (UAE)	5.5–39.1%	16.7%	Silverberg et al. [3]	Diagnosed AD, large population study.
		39.1%	Silverberg et al. [3]	Only ISAAC criteria, large population study.
		11.6%	Maspero et al. [4]	Large population study.
		5.5%	Al Hammadi et al. [10]	Dubai patients covered by private insurance.
		12.9%	Ibrahim et al. [9]	1944 children aged 6–7 years.
		14.6%	Ibrahim et al. [9]	1793 children aged 13–14 years.
		9%	Al-Hammadi et al. [10]	
		12%	Behbehani et al. [9]	
Saudi Arabia	12.5–45.4%	19.8%	Silverberg et al. [3]	Diagnosed AD, large population study.
		37.1%	Silverberg et al. [3]	Only ISAAC criteria, large population study.
		15.3%	Maspero et al. [4]	Large population study.
		45.4%	Alakeel et al. [6]	854 Taif citizens.
		12.5%	Al-Frayh et al. [8]	Overall prevalence of physician-diagnosed AD.
		43.5%	Al-Frayh et al. [8]	City of Hofuf.
		32.6%	Al Frayh et al. [8]	City of Riyadh.
		31.9%	Al Frayh et al. [8]	City of Jeddah.
		14%	Nahhas et al. [8]	Children aged 6–12 years.
Egypt	3.6–12.01%	3.6%	Maspero et al. [4]	Large population study.
		12.01%	Al Dhduh et al. [12]	308 students aged 11–14 years.
Kuwait	19.5%	19.5%	Ziyab et al. [11]	3775 adolescents aged 11–14 years.
Syria	3.9%	3.9%	Reda et al. [5]	ISAAC
Lebanon	11.5–11.8%	11.5%	Hallit, et al, Waked et al. [13,14]	5544 children/adolescents aged 5–14 years.
		11.8%	Waked et al. [15]	3909 children/adolescents aged 5–12 years.

## 3. Mechanisms

AD pathogenesis is multifactorial, and involves a complex interplay between the environment, genetics, the immune system, and other factors such as microbiome, which contribute to skin barrier dysfunction and inflammation [5,7,16]. Differences in AD seen in the Arab Middle East may be explained by a collection of these factors.

### 3.1. Environment

Environment plays a role in AD prevalence and symptoms, with factors such as climate, urban versus rural dwelling, pollution, diet, and comorbid conditions contributing to AD risk [7]. There is research to suggest that populations that migrate from areas with low prevalence of AD to areas of higher AD prevalence show an increased incidence of AD [17]. Moreover, climate conditions vary from country to country in the Arab Middle East and can influence AD prevalence and symptoms. AD prevalence is found to be increased in regions with dry weather, such as Saudi Arabia, which experiences a very dry and high-temperature desert climate [7,18]. The dry climate in such areas can exacerbate dry skin, contributing to the higher prevalence of AD [7]. However, conversely, there is a potential anti-inflammatory effect of the sun on eczematous lesions which may improve AD [19].

Dwelling in more urban environments has been linked with increased AD prevalence in the Middle East compared with rural environments. This is supported by large population studies encompassing countries in the Middle East, such as those conducted by Silverberg et al. and Maspero et al., which have demonstrated a higher prevalence of AD in urban areas compared to rural areas [3,4]. Urban environments are associated with greater socioeconomic status, higher education levels, increased air pollution, sedentary lifestyle and diet, and smaller family sizes, which are some factors that may explain the increased prevalence [4,7,20]. Differences in exposure to tobacco smoke, hygiene practices, rates of breastfeeding, exposure to infectious diseases, and various industrial and cosmetic antigens are likely to play a role as well [21]. Urban and suburban environments are also associated with improved living conditions, antibiotic use, and childhood vaccinations, resulting in fewer infections; as a result, the lack of microbial burden then redirects the typical Th1 immune response towards a Th2 response, predisposing individuals to a diverse array of atopic diseases—a concept termed the hygiene hypothesis [22].

There are behavioral factors and other environmental exposures to consider in Middle Eastern populations that may contribute to AD pathogenesis. One study found that waterpipe smoking by mothers is consistently and independently associated with atopic diseases in Lebanese children, including allergic rhinitis, asthma, and AD [23]. There is also evidence that exposure to tobacco smoke during pregnancy or infancy increases the risk of allergic rhinitis and asthma in early childhood and increases the risk of eczema at later ages [13]. A study by Alqahtani et al. found that potential risk factors associated with allergic diseases including AD include fast food consumption, trucks passing near houses, having a dog or cat at home, and a positive skin prick test for Bermuda grass or cat fur. However, it is important to acknowledge that certain findings, such as the potential influence of factors like “trucks passing near houses”, are limited due to the inherent difficulty in quantifying non-measurable variables, which may introduce variability and potential bias in the study findings [8]. A study from the Ahmed Maher Teaching Hospital in Cairo, Egypt found a significant association between the higher prevalence of allergic skin diseases including AD and the presence of animals at home, the presence of intestinal parasitic infection, and the intercurrence of other allergic phenomenon as asthma and allergic rhinitis [17].

### 3.2. Genetics and the Immune System

There is a growing body of evidence suggesting that there are ethnic differences in genetic and immune-related factors that are associated with an increased prevalence and incidence of AD [24]. The largest genome-wide association study to date in European, Japanese, African, and Hispanic populations identified 31 AD risk loci with variations among ethnic groups for many of these markers [21]. Genetic studies specifically on Arab Middle Eastern populations are limited. 

Among the genes implicated in the pathogenesis of AD are those involved in immune regulation and epithelial barrier function. Filaggrin is a central component of the skin barrier function and epidermal terminal differentiation, including the regulation of hydration in the epidermis and the pH of the skin. Loss-of-function mutations of FLG are associated with more persistent and severe AD, immune dysregulation, and increased rates of allergic sensitization and skin infections [1,21]. Although the Filaggrin gene has been shown to be the strongest and most consistent genetic risk factor for eczema development, research has shown that there are ethnic differences. It remains unclear what the role is of FLG mutations specifically in Arab Middle Eastern populations. In a study by Bogari et al., exome sequencing was carried out on Saudi Arabian children with AD and four mutations were discovered which were not previously connected with eczema [25].

Ethnic differences have been described in genes involved with innate and adaptive immunity, particularly with regards to Th2 signaling pathways. Upregulation of Th2 cytokines IL-4 and IL-13 has been shown to downregulate FLG expression, resulting in epidermal barrier dysfunction in the absence of FLG mutations. IL-4Ra genetic polymorphisms have been implicated in susceptibility to both atopy and asthma; moreover, an association between IL-4/IL-4Ra polymorphisms and susceptibility of AD has been described in Egyptians [21,26]. A genetic study by Hussein et al. found that in Egyptian children, IL-4Ra and STAT6 SNPs may play a role in susceptibility to AD. Additionally, gene–gene interactions between IL-4, IL-4Ra, and STAT6 significantly increase individual susceptibility to AD, potentially by influencing IgE production [26]. Further genetic studies are needed in Arab Middle Eastern AD patients to better understand specific ethnic differences.

### 3.3. Microbiome

Compared with healthy individuals, the skin of patients with AD is more susceptible to colonization by commensal organisms such as Staphylococcus, Malassezia, Candida, and Alternaria. These patients often develop allergic reactions to the weaker microbial protein antigens that would not elicit a response in healthy individuals with normal skin flora. Differences in skin microbiome in Arab Middle Eastern populations are likely to play a role in explaining differences in AD compared with other ethnic groups. Mohammed et al. reported the first study exploring the relationship between the skin microbiome and AD susceptibility in an Egyptian population and found that the bacterial diversity of the skin microbiome in patients with AD was less than those without AD. Genus-level analysis showed significant abundance variations by age, disease severity, locality, or immune response. *Streptococcus*, *Cutibacterium*, and *Corynebacterium* appeared to be specific signatures for AD in children, adolescents, and adults, respectively, and *Staphylococcus* was noted as a potential biomarker candidate for AD [27]. Interestingly, total IgE levels were found to be positively correlated with relative enrichment of certain *Staphylococcus aureus* subspecies, suggesting that skin microbiome differences in AD may be partly related to serum IgE levels [27]. Further microbiome studies in Arab Middle Eastern populations are a promising area of research.

There are also reports that qualitatively abnormal sweat is related to the condition of AD. The amount of antimicrobial peptide contained in sweat in patients with AD showed that the concentrations of LL-37 and dermcidin are related to the susceptibility to AD skin lesions [28]. These findings are likely to have implications for AD in subtropical areas of Arab Middle East. Whether the skin microbiome and/or the sweat content in Arab Middle Eastern AD patients differs from other ethnic groups or not remains to be elucidated. 

## 4. Clinical Differences

Most of the current literature on the ethnic differences in clinical presentations of AD is based on patients living in the United States or Europe, not from the original countries [29]. Overall, AD presents similarly across racial and ethnic groups; however, there are distinct clinical features to consider in Arab Middle Eastern patients. 

Depending on the region and country in question, darker skin tones are very common in the Arab Middle East. In darker skin types, erythema may appear more subtly as a darker red, red–brown, or violaceous, making it difficult to assess visually [29]. As a result, erythema may be missed on clinical examination and becomes less reliable in AD diagnosis in darker skin tones, as scoring systems that rely on erythema can underestimate AD severity in darker skin types, leading to delays in diagnosis and treatment, and the progression to more severe disease [21]. The pattern of AD lesions may also vary, with darker skinned patients more likely to present with extensor surface involvement as opposed to the predominantly flexural lesions that are seen in European Americans [21,29]. Itch may be more severe in darker skinned patients, and as a result of increased scratching, these patients commonly present with lichenification and prurigo nodules [21]. Other features more commonly seen in darker skin types include perifollicular accentuation, papulation, scaling, Dennie–Morgan lines, palm hyperlinearity, periorbital dark circles, and diffuse skin xerosis [16].

**Figure 1:**
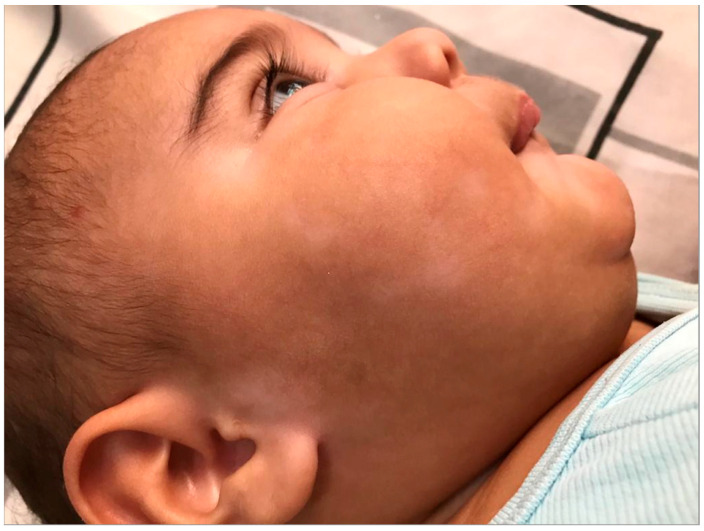
Steroid-induced hypopigmentation in an infant.

In the darker skin tones commonly seen in the diverse Arab Middle Eastern populations, pigmentary changes of the skin become an important concern. Patients with darker skin types are at higher risk for developing post-inflammatory dyspigmentation. Inflammation from AD and scratching can cause post-inflammatory hyperpigmentation that can last years without treatment. Chronic excoriation associated with severe AD may result in permanent dyspigmentation. Excessive sun exposure can worsen post-inflammatory hyperpigmentation, which is an important consideration in Middle Eastern patients considering the climate. Hypopigmentation is also a concern. The long-term use of potent topical steroids can lead to hypopigmented skin (Figure 1). Also, chronic scratching in the context of severe eczema can lead to hypopigmentation of the skin, which is often difficult to treat. In patients with darker skin tones, these pigmentary changes are often more noticeable due to the contrast with normal skin and can be very distressing to patients [16]. 

**Figure 2:**
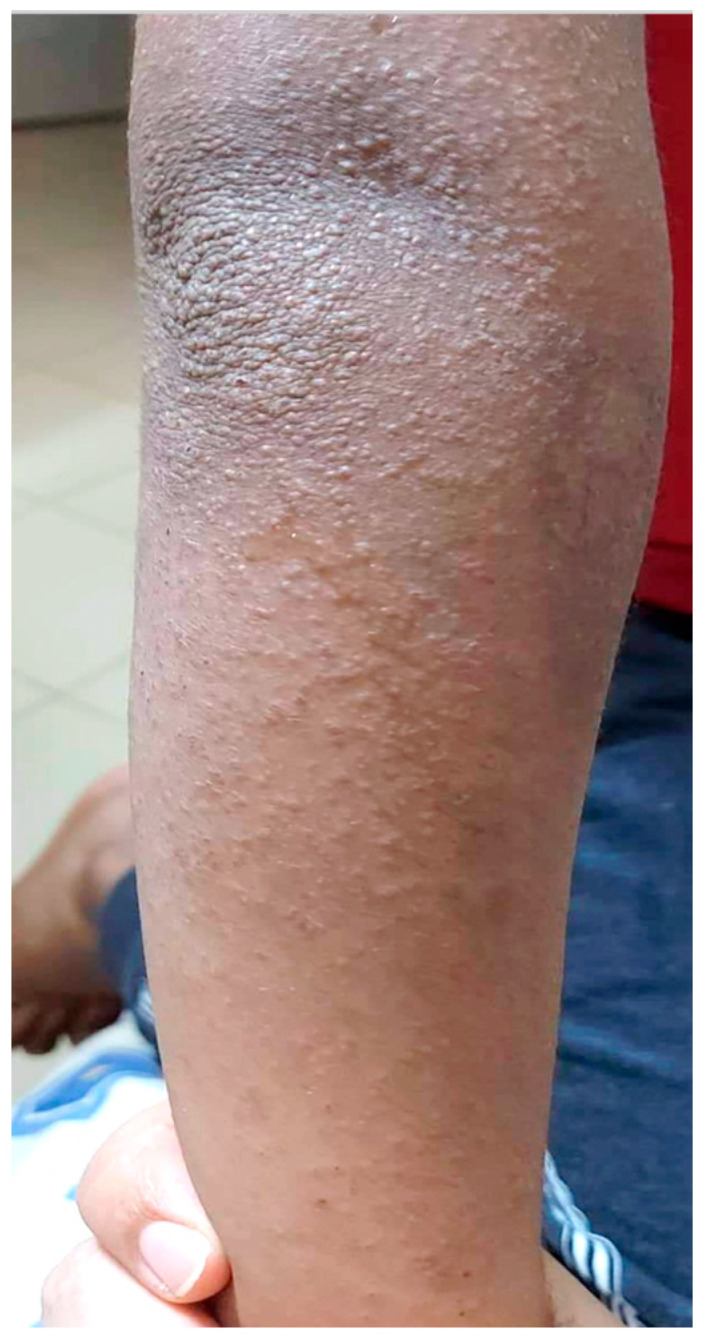
Eczematized miliaria in AD.

Climate and environment are additional important considerations, with hot, humid weather leading to exacerbation of AD with follicular or eczematized miliaria phenotypes (Figure 2). Heat and sweating increases itching, leading to scratching and worsening of eczema. Likewise, low-humidity environments can dry the skin and exacerbate AD lesions and itch, which is commonly seen in patients in Saudi Arabia due to the climate conditions.

**Figure 3:**
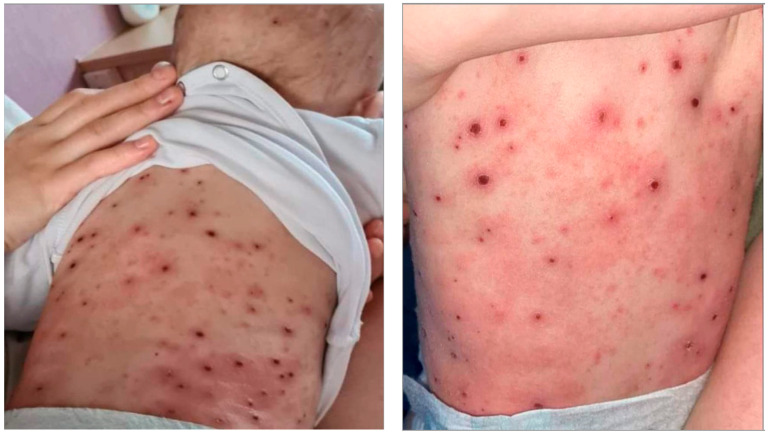
Kaposi’s varicelliform eruption.

Other phenotypes commonly seen are hand dermatitis, with variable severities, among kids, housewives, and working adults. Napkin dermatitis is also commonly seen in infants. Individuals of low socioeconomic classes are more prone to infections which may exacerbate and worsen their AD. Kaposi’s varicelliform eruption is reported in infants and children, where chicken pox vaccine is not compulsory, such as Egypt (Figure 3). 

**Figure 4:**
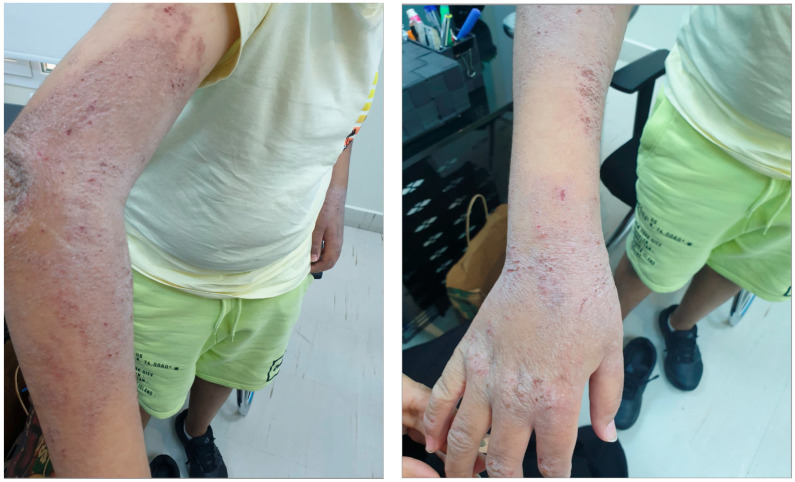
Severe pediatric AD.

**Figure 5:**
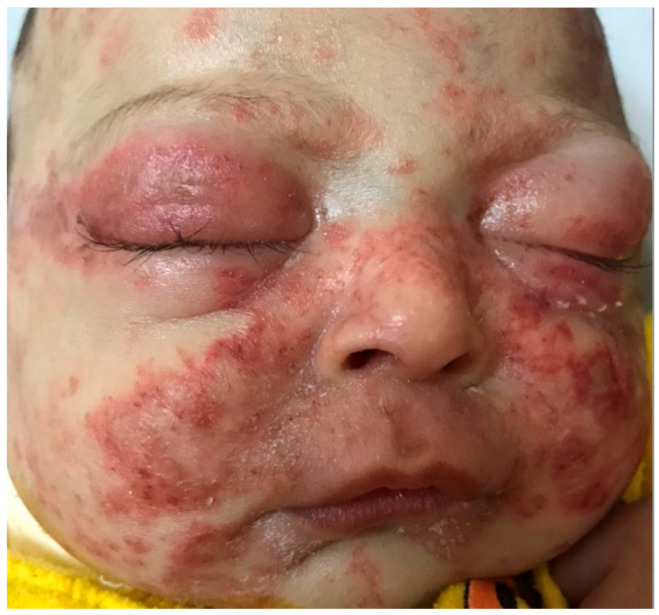
Severe infantile eczema.

Severe pediatric (Figure 4) and adult AD, for whom moderate-to-severe AD therapies such as biologic medications are unavailable or not covered by insurance, is a significant unmet need. It is of note that the hands (Figure 4) tend to show more severe disease symptoms and likely negatively impact social and professional life. Although the anti-IL-4 receptor alpha inhibitor dupilumab is now approved from 6 months onwards, infants below this age are still suffering (Figure 5). It is likely that in Arab Middle East countries involved in civil wars such as Yemen and Syria, AD and its management are not a medical priority and therefore patients lack access to appropriate therapy and hence they develop more severe forms of AD.

## 5. Disease Burden

AD patients in the Arab Middle East face a substantial economic and quality-of-life (QoL) burden that extends to their caregivers and families, as well as healthcare systems. The humanistic and economic burden differs from country to country, although it is significant in all countries. According to the Global Burden of Disease study, AD carries the highest burden of disability-adjusted life years (DALYs) among skin diseases worldwide; furthermore, the age-standardized rate of DALYs associated with AD surpasses that of other conditions such as liver cirrhosis [18]. Additionally, AD patients incur considerable personal suffering in comparison to many dermatologic and non-dermatologic conditions [30]. There are direct medical costs and also indirect costs incurred from lost time from work, and due to the chronic nature of AD, the cumulative costs can be substantial. There is also a considerable psychosocial cost from poor QoL. The economic and QoL burden of AD in the Arab Middle East has not been sufficiently quantified in the literature, especially in low- and middle-income countries where there is very little research on AD [31]. Furthermore, resource allocation is generally less prioritized in AD in comparison with diseases with high mortality because AD mainly affects QoL rather than the life years of patients [18]. Properly managing AD and addressing the unique challenges that arise in Arab Middle Eastern AD patients can help to decrease the associated burden, improve QoL, and reduce costs. 

### 5.1. Economic Burden

The Gulf area of the Middle East is considered to be more ethnically diverse, wealthier, and with more access to medical care for the nationals and the large number of insured immigrants. On the other hand, the Mediterranean part of the Arab Middle East shows less ethnic diversity, more economic difficulties, and unavailability of newer treatments, although some are approved. In addition, in the Mediterranean region, political circumstances and internal disputes have led to further economic problems as well as migration of nationals from Syria, Libya, Iraq, Palestinian territories, and Lebanon.

A study by Elezbawy et al. attempted to quantify the burden of AD in seven countries in the Middle East and Africa which included Egypt, Lebanon, Saudi Arabia, Kuwait, Algeria, South Africa, and the UAE. Burden was measured across many different parameters. They found that on average, a patient with AD loses 0.19 quality-adjusted life years (QALYs) or 19% of their health-related QoL from the disease, which is comparable to more severe conditions such as kidney transplantation. QALY loss was reported to be higher in countries with greater population sizes such as Egypt, and less in countries with smaller populations such as Kuwait [18].

Higher-income countries, which are typically countries in the Gulf area, have the highest AD-related healthcare expenditures. Annual healthcare costs per person were found to correlated with the country’s socioeconomic status and cost of healthcare services, as seen with the UAE (USD 3569 per person annually) and Kuwait (USD 2880 per person annually). This is likely due to the fact that in higher-income countries such as the UAE and Kuwait, medical therapies are relatively more expensive, and more advanced treatments such as targeted therapies are often more available and frequently used. At the country level, annual healthcare costs were highest in the UAE (USD 112.5 million), in which all nationals and at least 80% of residents are under health insurance, with the second and third highest being Saudi Arabia (USD 99.5 million) and Egypt (USD 95.5 million); the lowest reported was Lebanon (USD 13.6 million). These costs correlated with population and income level. The included countries allocated between 0.20% to 0.77% of their healthcare spending to AD-related expenditures [18].

Indirect costs significantly contribute to the costs of AD burden. The literature shows that on average each patient with AD loses approximately 28.9 days of productivity annually because of the disease. At the country level, Saudi Arabia loses approximately USD 364 million annually due to indirect costs associated with AD, and the UAE loses about USD 228 million. Kuwait, Egypt, and Lebanon had much lower estimates, ranging from USD 33 million in Lebanon to USD 62 million in Kuwait. On average, the indirect costs associated with AD represent around 0.043% (range of 0.022% to 0.059%) of the gross domestic product of each country. This accounts for approximately 67% of the total costs, ranging from 37% in Egypt to 79% in Saudi Arabia, highlighting the significant effect of indirect costs associated with AD. When the QALYs lost due to AD were converted to a monetary value, the humanistic burden was then reported to be about 2.4 times greater than the total economic burden in all of the countries. This highlights the considerable hidden cost of AD, in addition to the tangible, direct costs due to the disease [18].

Moderate-to-severe treated AD is associated with the highest costs, as demonstrated by a UAE study reporting the per-patient gross cost for moderate-to-severe treated AD to be USD 531.5, compared to mild-to-moderate treated AD (USD 378.38) and untreated AD (USD 144.05) [10].

Atopic comorbidities such as allergic rhinitis, asthma, and food allergies are also important to consider and can drive up costs [5,10]. A child with moderate-to-severe AD can have up to a 50% risk of developing asthma and a 75% risk of developing hay fever [32]. An Egyptian study of 308 children found the prevalence of asthma and allergic rhinitis to be 46.1% and 12.33%, respectively [12]. A Lebanese study found that 39.6% of children between ages 5 and 14 are affected by allergic diseases, which include asthma, allergic rhinitis, and AD [14,15]. These findings highlight the high prevalence of atopic comorbidities. Other comorbid conditions associated with AD include cardiovascular disease; metabolic diseases such as obesity, malignancy, and gastrointestinal problems; and psychological disorders. Atopic and other comorbid conditions contribute to the burden of AD, and are important to consider in AD in both children and adults in the Arab Middle East.

### 5.2. QoL Impact

AD has a substantial and multidimensional impact on patients and their families and caregivers, with effects on physical, emotional, social, and economic components of family life [31]. There is a significant effect on patient QoL, with itch, anxiety and depression, and sleep disturbances being the most frequently reported in patients [1,33]. The prevalence of AD is higher in younger age groups, with data showing AD burden is highest in patients aged 1 to 5 years [22]. Children can experience physical discomfort from persistent itching and scratching, which can lead to sleep disturbances with effects on concentration, functioning, and productivity at school. Children can also feel embarrassment or face bullying due to their skin condition, leading to low self-esteem, social isolation, and mental health problems [7]. Adolescents and young adults face a similar QoL impact.

A study by Alzolibani performed in the Dermatology Clinics and Hospitals affiliated to Qassim University, Buraidah, Saudi Arabia found that itching and scratching had the greatest impact on QoL in children with AD followed by the child’s mood and the time it took for the child to get to sleep [34]. Saudi Arabian QoL studies shed light on the multidimensional, negative QoL impact Saudi Arabian patients experience due to their skin condition. Data from 283 Saudi Arabian patients with skin diseases who completed the Arabic Skindex-16 QoL assessment tool, which is adapted for and validated in Saudi Arabian individuals, found eczematous dermatitis to be associated with the highest mean symptom domain score, indicating the highest physical burden of disease in patients with AD [7]. Another QoL study in Saudi Arabian patients found that 31.4% of patients had comorbid conditions, with around 20% of patients reporting psychiatric comorbidities [7]. This aligns with other research assessing the relationship between psychiatric and dermatologic conditions in the Middle East. The physical and psychosocial impact of AD contributes to the higher prevalence of psychiatric comorbidities seen in AD patients. A 2016 study of 254 adult and teenage Saudi Arabian patients with dermatologic conditions including AD reported overall prevalence rates of 7.5% for stress, 12.6% for depression, and 22.1% for anxiety. Among the patients, 56 had AD with prevalence rates of 7.1% for stress, 10.7% for depression, and 21.4% for anxiety, which is similar to the overall pool of patients with dermatologic conditions. Another study of 875 Saudi Arabian dermatology patients estimated an overall prevalence of 14% for depression and 29% for anxiety [7].

Another study by Hossny et al. found that of 85 children with AD from the children’s hospital in Ain Shams University in Egypt, more than half of the children (55.4%) had severely affected QoL, with itching and pain being a primary concern. A statistically significant effect of face eczema on QoL of children was also reported [35,36]. In this study, 84.6% of the children believed that the disease had a great impact on their relationship with friends and play, while this was reported in only 2% by other investigators, highlighting the social stigma that patients may feel. 

A Kuwaiti study in adolescents aged 11 to 14 years reported that a significant proportion of the adolescents had nocturnal sleep disturbances due to itchy rash [11]. In a UAE study, 6.7% of children aged 6 to 7 years and 12.4% of adolescents aged 13 to 14 years experienced an itchy rash that woke them more than one night a week [9]. The study included school class registers residing in Dubai and the Northern Emirates and did not define the studied ethnic groups nor specify how the questionnaire was filled, at least with younger children of 6 to 7 years old without a guardian. Therefore, these figures are likely to be less than expected.

The burden of AD extends to families and caregivers, as they often have to care for the child, with time lost from work, added expenses, and increased stress. Parents often report fatigue owing to care-related loss of sleep, emotional burden, and feelings of frustration, stress, and confusion [7]. There are few studies evaluating the impact of AD on parents and caregivers of children with AD in real-world populations. One study found that factors such as sleep disturbance, increased monthly expenditure, and emotional distress to be commonly reported, all of which are found to worsen with higher disease severity [5]. Living with a child with AD may have economic implications resulting from missed workdays, as well as impaired productivity while at work [31]. Sleep problems, in particular, were reported to be a factor that may exacerbate other effects associated with daily management of a child with AD, such as tiredness, fatigue, and psychosocial stress. Additional data using the Arabic version of the Dermatitis Family Impact Questionnaire suggest that the QoL of Saudi Arabian families is substantially affected when a child or infant has AD. A study of 447 Saudi Arabian children and infants with AD found that 3.4% of families had a normal QoL, whereas 66.4% experienced a moderate impact on QoL [7]. A small-scale cross-sectional study evaluating the QoL of Egyptian parents of 100 children with AD (67% with severe AD) found that AD directly affects the QoL of patients, and that a negative QoL impact significantly correlated with disease severity [35]. The study used the validated Arabic version of the Dermatitis Family Impact (DFI) questionnaire and found that the highest-scoring DFI domains were sleep time, followed by a feeling of tiredness, emotional stability, general life, household work, and expenditure [35].

It is burdensome to care for a child with AD because of the special needs of the patient, such as with bathing, wet dressings, topical application, and household-related tasks and responsibilities such as food restrictions, clothing selection, laundry, and house cleaning to avoid potential allergens [36]. This is in addition to the added costs of implementing these lifestyle adjustments [1]. In the Egyptian study by Abdel-Maguid et al., it is interesting to note that leisure activity, shopping, relationships among family members, and distribution in preparation of food were amongst the lowest-scoring items, which is a testament to the strong family relationships that are often a hallmark in Egyptians and other Arab Middle Eastern communities [35]. The study authors postulated that the female predominance of AD (58%) could be attributed to the special care that female children often receive in these families, with families more likely to feel anxiety and distress over marriage and post-marriage relationships. This is a unique burden to women in the Middle East, who are more likely to face social and marital concerns of suffering with moderate-to-severe AD. 

These studies highlight the significance of addressing the psychosocial well-being of AD patients as well as considering the family and caregiver impact in the management of AD.

## 6. Unmet Needs and Recommendations

There are many unmet needs with regards to AD in the Arab Middle East—some of the most important of which are lack of data and research, patient and provider education, consistent guidelines, continuity of care and access to treatments, and research into treatment safety and efficacy specifically in Arab Middle Eastern patients. 

### 6.1. Data

Scarce literature and a lack of regional data on AD in the Arab Middle East are large barriers to effective care. The Arab Middle East is very heterogenous with regards to socioeconomics, healthcare infrastructure, clinical practice, and access to care, and with limited regional data, it becomes difficult to understand the full scope of unmet needs. This is particularly an issue in lower socioeconomic areas where resources and information are more limited.

Across the Arab Middle East, there is a need for local and regional epidemiologic data on AD, data on clinical and phenotypic differences, and burden data that address not only the direct healthcare costs of AD, but also indirect costs and impact such as with lost productivity and diminished QoL of patients and caregivers. Additionally, region-specific burden data on AD symptoms such as itch intensity would be helpful, although they would necessitate the use of tools such as the standardized itch questionnaire [7]. These studies should address the local healthcare systems and the sociocultural characteristics of the populations. Access to more country-specific burden data will allow for evidence-based decisions moving forward with the proper allocation of resources to these areas [18].

As information is collected, the methodology and reporting of the data are important to consider. There may be misclassification bias and inaccurate reporting of information into databases, which was reported as a study limitation of a UAE study by Al Hammadi et al. [10]. Hanifin et al. estimated that 37.1% of AD cases are diagnosed by a physician, meaning that there is a large population of potentially untreated AD patients which will not contribute to healthcare costs, resulting in underestimation of the burden [18]. Measuring of epidemiologic data such as prevalence and incidence is difficult with varying methodologies making it hard to compare studies (is the AD diagnosis self-reported or formally diagnosed?), and it becomes difficult to quantify QoL data and indirect costs for similar reasons. Tools to effectively measure QoL are limited. For example, Arabic versions of the DLQI have been validated in Moroccan dermatology patients, but these versions of the DLQI have not been specifically validated for AD patients or other Middle Eastern populations [21]. It is important when administering outside questionnaires that they are translated to fit the local colloquial Arabic terminology, while ensuring that the content is accurate and comprehensible, and then to have these questionnaires validated by experts [8,9]. Cultural differences in survey responses and disparities in access to healthcare may influence data collection as well and are important elements to consider.

### 6.2. Healthcare Systems and Cost

The chronic, relapsing nature of the disease presents a large financial burden to patients, with more severe AD often requiring biologic and systemic therapies that are expensive and difficult to acquire in certain countries. Patients with moderate-to-severe disease requiring targeted therapies may decline treatments due to cost or if insurance coverage is not available. Regional differences in accessibility to specialized dermatologists, care availability, and cost further compound the situation; and in less affluent countries and with patients with lower socioeconomic status, there will be challenges with access and continuity of care and treatment adherence [2,29]. In many regions of the Middle East, healthcare systems are limited and ineffective. These factors add to the already significant psychosocial impact of AD and contribute to the deterioration of patient QoL. Even mild AD can incur significant costs, which can exacerbate financial insecurity in AD patients and their families, with problems in paying medical bills and accessibility to specialists and delays in appointments and treatments due to cost and not being able to afford treatments.

### 6.3. Guidelines and Treatment Availability 

In the Arab Middle East, there is a lack of consistent diagnostic and management guidelines that consider regional risk factors and differences in climate and environment, socioeconomics, treatment availabilities, diet and lifestyle, and patient skin types, as well as integrating local cultural and treatment practices. Current guidelines derived from European and North American populations may not be suitable for Arab Middle Eastern patients [2]. Biologic medications and other newer therapies referenced in existing AD guidelines may not be approved in the country of interest [6]. Therefore, it is essential to develop guidelines specifically tailored to the Arab Middle Eastern population to address these disparities.

A study by Ammoury et al. evaluated the management strategies of physicians in Egypt, Lebanon, UAE, and Saudi Arabia and found that there is a significant variation in types of treatments used, referral patterns, and treatment familiarity [2]. This study highlights the lack of a standardized approach to AD management in many countries in the Middle East. Many of the surveyed physicians were also unfamiliar with many of the newer AD treatments, such as crisaborole, or inadequately utilized treatments [2]. Previous studies have shown that patients with AD in this population often receive ineffective or inappropriate care, such as oral antihistamines, oral corticosteroids, or traditional medicines and remedies. When treating pruritus, the majority (74%) of physicians often prescribed antihistamines, which are demonstrated to have little efficacy in the treatment of AD. A study by Al Hammadi et al. found that in Dubai there was a substantial usage of antihistamines in treating AD [10]. Al Hammadi et al. also found that although 90% of AD patients are being treated by a specialist, there is significant underuse of newer treatments including biologic medications [10]. These studies demonstrate the need for consistent guidelines and increasing physician awareness and education on AD treatments, especially as new treatments become available in the Arab Middle East.

Specialist education should be tailored to the specific populations they serve, considering variations in clinical presentation, and addressing diagnostic dilemmas associated with differential diagnoses such as immunodeficiency syndromes and zinc deficiency [1]. A large population study by Maspero et al. found that in Saudi Arabia, general practitioners were the most common diagnosing physicians of AD [4]. Therefore, it is important that general practitioners also receive training and education in dermatology [7]. Training sessions, conferences, and social initiatives geared towards increasing awareness of AD can help to improve physician knowledge. This will help to alleviate delays in diagnosis and appropriate treatment. 

There is a need for further studies on the effectiveness of different management strategies on AD patients in the Arab Middle East, and eventually the development of regional standardized guidelines for the treatment of AD [2]. These guidelines should consider the unique characteristics of the region. For instance, in tropical and subtropical areas, factors such as the occlusion of ointments and emollients and the feasibility of wet wrap therapy in hot and humid climates should be considered [29]. They should also consider the cultural practices of the region, as some may worsen AD symptoms [16]. 

Ashraf et al. provided the first proposed topical treatment guidelines specifically developed for AD patients in the Arab Middle East [5]. The guidelines were developed by a panel of six experts in the Middle East and one in Europe and was based on a review of published international and national guidelines on AD, an evaluation of relevant literature published up to August 2016, and local treatment practices. The algorithm proposes the use of the topical calcineurin inhibitor pimecrolimus 1% cream twice daily until clearance for acute-to-moderate AD flares on sensitive body areas (face, neck, genital area, and inguinal folds). For other, less sensitive body areas, the use of a topical calcineurin inhibitor—pimecrolimus 1% cream, tacrolimus 0.03% ointment in children, or 0.1% ointment in adults—was recommended to be applied twice daily until clearance. For severe acute AD flares, a short course of topical corticosteroids was recommended prior to the use of the above indicated topical calcineurin inhibitors until resolution of lesions [5]. 

Access to novel, effective therapies such as biologics can significantly improve patient QoL [37]. Even though the phosphodiesterase-4 inhibitor crisaborole (for mild-to-moderate AD) and the anti-IL-4 receptor alpha inhibitor dupilumab (for moderate-to-severe AD) have been recently approved in Egypt, Lebanon, the UAE, and Saudi Arabia, cost and accessibility are limitations to use [2,7]. Dupilumab has proven to be a safe and effective treatment for moderate-to-severe AD, with recent studies showcasing its long-term efficacy, psychological advantages, and rapid relief of pruritus. Improving the accessibility of dupilumab in these regions would immensely benefit patients, providing them with improved outcomes and quality of life [38,39,40,41]. Likewise, the oral JAK inhibitors barcitinib (approved in Egypt), abrocitinib (approved in Egypt and the UAE), and upadcitinib (approved in the UAE and Saudi Arabia), as well as the anti-IL-13 monoclonal antibody Tralokinumab (approved in the UAE), have been approved for the treatment of moderate-to-severe AD. However, their prescription has been limited thus far [2].

### 6.4. Patient Education and Care

In addition to training medical providers on how to effectively diagnose and manage AD, educating patients and their families and caregivers on the disease, treatment options, and the appropriate use of medications is essential to facilitating adherence and effective long-term care. 

Corticosteroid phobia is prevalent in Middle Eastern communities and can adversely affect adherence and treatment outcomes for AD patients. This fear is often rooted in concerns about hypopigmentation caused by prolonged corticosteroid use, leading to underutilization or nonuse of prescribed topical corticosteroids [29]. Patient education on the safe use of corticosteroids and exploration of alternative therapies by physicians are important strategies to address corticosteroid phobia and ensure effective treatment of AD [2,5]. 

It is important to provide patients and caregivers with education on effective skin hygiene strategies. This includes guidance on selecting appropriate skincare products, clothing, and laundry items. Opting for high-quality, hypoallergenic, and fragrance-free products is recommended, although their cost and lack of insurance coverage may pose challenges [32]. When choosing skincare products, it is advisable to prioritize those with clinical efficacy in improving the skin barrier, while also considering their effectiveness and affordability within the region [32].

When discussing AD management with patients, socioeconomic status, level of education, culture, and the local healthcare system should be considered.

Comprehensive patient education encompasses the discussion and effective management of comorbid conditions and identifying allergies and triggers, which can lead to significant cost reductions, benefiting both patients and healthcare systems. It is important to note that environmental allergens vary across different regions in the Arab Middle East, necessitating region-specific assessments [8]. Diagnostic tests developed for European and North American populations, such as the skin prick test for IgE immunoreactivity, may not be as relevant for Arab Middle Eastern populations [7].

Implementing and encouraging lifestyle changes is integral to AD management, and promoting an anti-inflammatory lifestyle can help to improve the disease. Emphasizing the importance of maintaining adequate sleep, adopting a healthy diet, and practicing stress management techniques can have a positive impact on AD symptoms and overall well-being. Health education programs can help patients and their families adapt to the different challenges they will face with managing AD [36]. Programs such as eczema schools that involve families, healthcare providers, psychologists, and dieticians can provide extremely helpful information to Arab families regarding coping mechanisms, stress management, and daily care of eczema [32].

### 6.5. Future Research

Studies are needed to assess the treatment efficacy, safety, and cost-effectiveness of new and existing therapies in the Arab Middle East. Ethnic differences in genetic and immune profiles have been documented and may impact treatment response [2,7]. There is a need for further real-world studies specifically in Arab Middle Eastern populations, as they may provide valuable information to guide treatments and guidelines. Special consideration and global support should be allocated to Arab Middle East countries with political circumstances, internal disputes, and economic difficulties.

## 7. Conclusions

Treating and managing AD in the Arab Middle East is challenging, with many barriers to effective care and a lack of region-specific data. The chronic, relapsing nature of AD, in conjunction with barriers to care, presents a significant humanistic and economic burden to patients and caregivers. Mechanisms behind the ethnic differences of AD in the Arab Middle East involve an interplay of environment, genetics, and immune system. Tailoring management guidelines to account for region-specific factors including environment, sociocultural practices, and socioeconomics; increasing patient and provider education; and improving access to care will help to alleviate some of the burden. Further studies exploring molecular biomarkers, genetic polymorphisms, immune factors, and the microbiome of patients in the Arab Middle East will help to elucidate the mechanism behind ethnic differences in AD in this population as well as help to understand treatment response and susceptibilities.

## Data Availability

Not applicable.
